# Trajectory of frailty among the older people in a community setting: A cohort study from the Shizuoka Kokuho Database

**DOI:** 10.1007/s40520-026-03434-z

**Published:** 2026-06-14

**Authors:** Shiori Nishimura, Kazuki Morohoshi, Ryo Nakamaru, Hisateru Tachimori, Hiroyuki Yamamoto, Eiji Nakatani, Masato Takeuchi, Yoshiki Miyachi, Shun Kohsaka, Hiroaki Miyata, Hiraku Kumamaru

**Affiliations:** 1 Shizuoka Graduate University of Public Health, Shizuoka Shizuoka, Japan; 2 Department of Healthcare Quality Assessment, The University of Tokyo Graduate School of Medicine, Bunkyo, Tokyo Japan; 3 Department of Health Data Science, Yokohama City University Graduate School of Data Science, Yokohama, Kanagawa Japan; 4 Department of Health Policy and Management, Keio University School of Medicine, Shinjuku, Tokyo Japan; 5 Department of Cardiology, Keio University School of Medicine, Shinjuku, Tokyo Japan

**Keywords:** Frailty trajectories, Electronic frailty index, Oldest-old, Sex differences, Healthy longevity

## Abstract

**Background:**

Long-term population-level evidence on frailty trajectories, particularly among the oldest-old, remains limited.

**Aims:**

To evaluate 6-year frailty trajectories in community-dwelling adults aged ≥ 50 years.

**Methods:**

We conducted a cohort study using a regional Japanese administrative claims database from 2012 to 2020. Frailty was measured using the electronic Frailty Index. Inverse probability of censoring weighting accounted for insurance-plan disenrollment, and weighted multinomial logistic regression estimated adjusted 6-year transition probabilities.

**Results:**

The cohort included 1,040,663 adults aged ≥ 50 years (mean age, 73.2 years; 58.6% women). Six-year final status worsened with baseline frailty severity: death increased from 12.7% among fit individuals to 23.8%, 34.7%, and 49.1% among those with mild, moderate, and severe frailty. Among those with severe frailty, recovery to fit status occurred in 2.6%, and recovery to fit or mild frailty in 9.0%; 91.0% died or were in moderate-to-severe frailty. Age- and sex-specific patterns differed. Among fit adults aged 65–74 years, women had lower mortality than men (4.3% vs. 8.7%) and higher probabilities of being alive with mild or moderate frailty (26.6% vs. 21.8%; 6.1% vs. 4.9%). Among fit adults aged ≥ 85 years, death was the dominant final state, although women had lower mortality (53.9% vs. 59.0%) and higher fit status (21.7% vs. 18.8%).

**Discussion:**

Severe frailty was rarely followed by recovery to fit or mild status. Sex differences in frailty trajectories varied across age groups.

**Conclusions:**

These findings highlight severe frailty as a late high-risk state and may inform age- and sex-specific frailty risk stratification and care planning.

**Supplementary Information:**

The online version contains supplementary material available at 10.1007/s40520-026-03434-z.

## Introduction

Frailty, characterized by increased vulnerability to stressors and adverse outcomes such as falls, disability, hospitalization, and mortality, has become a major concern in aging populations worldwide [1–3], including Japan [4, 5]. Two primary assessment models, phenotype and cumulative deficit models [2, 6, 7], exhibit moderate predictive ability for outcomes such as mortality and hospitalization [6, 8]. The electronic frailty index (eFI) is based on cumulative deficit model, developed by Clegg et al. in the UK [9], and the UK NHS recommends its use for identifying frail older adults in routine clinical care [9–11]. The eFI demonstrated its utility in predicting mortality and utilization of caregiving services among elderly individuals in the community-dwelling older adults.

As frailty is dynamic and continuous [12–14], repeated measurements of frailty using eFI hold significant importance for identifying groups that show improvement in frailty. Identifying a group with the potential to intervene in preventing advanced frailty is crucial for health policy planning in community settings [15]. Previously, Gill et al. reported that the temporal changes in frailty over 4.5 years, with 58% experiencing either a reversal or progression in their frailty status during follow-up [13]. Additionally, advanced age and being women were identified as factors associated with worsening frailty, and those aged 75 and above tend to have lower frailty reversibility [16]. These findings underscore the importance of developing administrative policies that account for the dynamic nature of frailty.

Demographic characteristics, particularly age, are important determinants of frailty and should be considered when evaluating frailty transitions over time. Japan is one of the most rapidly aging societies worldwide, with approximately 30% of its population aged 65 years or older [17, 18], including 5.5% of the oldest-old (aged ≥ 85 years), and is often described as a “super-aged society”. By contrast, globally, nearly 10% of the population is aged ≥ 65 years and about 2% is aged ≥ 80 years [19], reflecting ongoing population aging worldwide. Additionally, in Japan, where life expectancy is among the highest in the world (84.5 years), healthy life expectancy remains considerably shorter (73.4 years) [20]. Therefore, assessing frailty trajectories in Japan, particularly among the oldest-old, may provide useful insights into healthy longevity, not only for Japanese health policy but also for other countries now undergoing population aging.

Japan’s healthcare system has internationally distinctive features, including a combination of universal health insurance and a public long-term care insurance system for older adults [21]. Population-level evaluation of long-term frailty trajectories within Japan’s healthcare and long-term care systems can provide real-world evidence on frailty progression, recovery, and death under routine care conditions, and may inform risk stratification, prevention strategies, and health policy planning in advanced aging societies. In this study, we aimed to investigate temporal changes in frailty trajectories in a large community-based older population in Japan.

## Methods

### Study design and data source

We conducted a population-based cohort study using the Shizuoka Kokuho Database (SKDB), an administrative database covering residents of Shizuoka Prefecture, Japan, incorporating more than two million enrollees from April 2012 to September 2020 [22]. The source population of this study comprised all individuals enrolled in the SKDB during the study period. It was therefore restricted to beneficiaries of the National Health Insurance (NHI) and Late-Stage Elderly Medical Care System (LSEMCS) in Shizuoka Prefecture.

The SKDB integrates monthly health insurance claims from the NHI (mainly self-employed individuals and retirees aged ≤ 75 years) and the LSEMCS (all residents aged ≥ 75 years), together with subscriber list (including age, sex, date of death and reasons for disenrollment), medical fee claim data (disease diagnoses, drug dispensation, procedures, device utilization), annual health checkup data, and long-term care insurance claims. All data are linked using a unique individual identifier. The coverage for residents in SKDB varies slightly between men and women, with approximately 20% among those aged 50–59 years, 40–50% among 60–64 years, 70–80% among 65–74 years, and nearly 100% among ≥ 75 years [22]. Consequently, the eligible population for this study reflects the demographic structure of NHI and LSEMCS beneficiaries rather than the entire prefectural population. The study was approved by the Ethics Committee of Shizuoka Graduate University of Public Health (Shizuoka, Japan) [(#SGUPH_2021_001_006].

### Participants

We included all beneficiaries registered in SKDB as of April 1, 2014. We designated April 1, 2014 as the index date, and defined the look-back period as the preceding 12 months from the Index date. Eligibility criteria included residents aged 50 and older at the index date and continuous enrollment in the health insurance plans during the entire look-back period (Supplementary Fig. 1).

### Frailty measurements

Frailty was assessed using the electronic Frailty Index (eFI), based on the cumulative deficit model proposed by Clegg and colleagues. This eFI comprises 36 equally weighted deficits identified in an individual, which are recorded using the Clinical Terms Version 3 Read codes routinely gathered in primary care data in the United Kingdom (Supplementary Table 1) [9, 23]. In the present study, frailty was not directly assessed through a dedicated clinical frailty evaluation. Instead, the eFI was constructed using routinely collected administrative claims data from the SKDB.

Each deficit was identified based on diagnostic codes and relevant information recorded during usual medical care. Thus, deficits were considered present if corresponding diagnoses or conditions were recorded in the claims data during the defined look-back period. We used the converted version of the deficit variables to International Statistical Classification of Diseases and Related Health Problems 10th Revision (ICD-10) codes based on previous studies [24, 25]. The eFI was then calculated by observed deficits as a numerator and the total number of deficits as a denominator, which is 35 since “fall” was not available in Japanese disease coding system [8] and therefore structurally missing for all participants. Therefore, the eFI score was calculated as the proportion of deficits present out of the total 35 deficits (e.g., 6/35 = 0.17). This approach aligns with the cumulative deficit model underpinning the eFI, which permits variation in the number of included deficits when data availability differs [26].

“Polypharmacy” was defined as being prescribed ≥five drugs over ≥ six months during the assessment period [27]. The eFI for each patient was calculated, and we categorized the participants into groups, using the cutoff points described in the original studies: fit (0-0.12), mild frailty (> 0.12–0.24), moderate frailty (> 0.24–0.36), and severe frailty (> 0.36) [9]. Frailty was assessed at four time points, spaced two years apart, from April 1, 2014, to April 1, 2020. Each assessment period covered the preceding 12 months, which we used to determine the eFI for each individual (Supplementary Fig. 2). The severity of frailty was classified, and the transitions were described over time.

### Statistical analysis

We tabulated the baseline characteristics of the participants. The individuals were classified into the following age groups: 50–64, 65–74, 75–84, and 85 years and above. A density curve and histogram were depicted to present the distribution of eFI.

Frailty transitions and mortality proportions were depicted using Sankey diagrams. We also conducted subgroup analyses stratified by age group. Individuals who left the insurance plan for reasons other than death were categorized as loss to follow-up. In addition, sex-stratified analyses were performed, and predicted probabilities of frailty status at 6 years after follow-up initiation were estimated. To account for potential bias due to disenrollment during follow-up, stabilized inverse probability of censoring weighting (IPCW) were estimated using models incorporating baseline age, sex and time-varying frailty category, and IPC-weighted Sankey diagram was constructed. We then estimated IPC-weighted predicted probabilities and adjusted odds ratios with 95% confidence intervals for frailty status at 6 years using a multinomial logistic regression model that incorporated sex, age group, baseline frailty category, and their interaction terms.

The Sankey diagram was created using the networkD3 package in R version 4.1.1 (R Foundation for Statistical Computing, Vienna, Austria), and other statistical analyses were performed using SAS version 9.4 (SAS Institute, Cary, NC, USA).

## Results

### Participant characteristics and baseline frailty

Among the 1,040,663 individuals included for analysis, the number of enrolees in each age group was as follows: 50–64 years: 211,945 individuals (20.3%), 65–74 years: 359,811 individuals (34.6%), 75–84 years: 312,888 individuals (30.1%), and 85 years and older: 156,019 individuals (15.0%). The baseline eFI had a heavily right-skewed distribution, as 178,063 (17.1%) of participants’ eFI scores were zero, with a median (25th and 75th percentiles) of 0.11 (0.03, 0.20) and a maximum of 0.77. Based on the eFI, 556,580 (53.5%) were categorized as fit, 289,963 (27.9%) as mildly frail, 141,983 (13.6%) as moderately frail, and 52,137 (5.0%) as severely frail.

Among deficits used in eFI, hypertension was most frequently observed (534,683, 9.6%), followed by peptic ulcer (*n* = 478,326, 8.6%), and diabetes (*n* = 407,489, 7.3%) (Table 1). The baseline characteristics are also presented for individuals aged 85 and older (Supplementary Table 2). Compared to the overall population, the proportion of those categorized as fit decreased among the 85 and older group, while the proportions of those classified as mild, moderate, and severe increased. Regarding the deficits used to calculate the eFI, hypertension was most frequently observed (*n* = 111,491, 8.9%), followed by polypharmacy (*n* = 94,477, 7.6%), peptic ulcer (*n* = 91,851, 7.3%). Other prevalence of deficits showed a different trend compared to the overall population (Supplementary Table 2). Among individuals who were neither dead nor lost to follow-up, the distribution of eFI shifted to the right over the following six years (Supplementary Fig. 3).

**Table 1:** Baseline characteristics

Characteristics			Frailty status							
	Total, *n* (%)		Fit, *n* (%)		Mild frailty, *n* (%)		Moderate frailty, *n* (%)		Severe frailty, *n* (%)	
	1,040,663	(100)	556,580	(100)	289,963	(100)	141,983	(100)	52,137	(100)
Age (years), n (%)										
50–64	211,945	(20.4)	172,417	(31.0)	30,872	(10.6)	7,038	(5.0)	1,618	(3.1)
65–74	359,811	(34.6)	227,482	(40.9)	94,255	(32.5)	30,501	(21.5)	7,573	(14.5)
75–84	312,888	(30.1)	114,814	(20.6)	110,218	(38.0)	63,771	(44.9)	24,085	(46.2)
≥ 85	156,019	(15.0)	41,867	(7.5)	54,618	(18.8)	40,673	(28.6)	18,861	(36.2)
Women, n (%)	610,016	(58.6)	306,691	(55.1)	176,968	(61.0)	91,823	(64.7)	34,353	(65.9)
Deficits in the electronic Frailty Index, n (%)										
Activity limitation	57,828	(1.0)	5,419	(0.5)	17,092	(0.8)	19,061	(1.2)	16,256	(2.0)
Anaemia and haematinic deficiency	149,668	(2.7)	26,884	(2.4)	51,561	(2.5)	44,351	(2.8)	26,872	(3.3)
Arthritis	271,699	(4.9)	64,348	(5.8)	101,998	(5.0)	72,197	(4.6)	33,156	(4.0)
Atrial fibrillation	138,791	(2.5)	19,721	(1.8)	49,359	(2.4)	44,195	(2.8)	25,516	(3.1)
Cerebrovascular disease	234,200	(4.2)	38,208	(3.5)	87,191	(4.3)	72,313	(4.6)	36,488	(4.4)
Chronic kidney disease	62,550	(1.1)	9,011	(0.8)	19,360	(0.9)	19,516	(1.2)	14,663	(1.8)
Diabetes	407,489	(7.3)	114,067	(10.3)	157,313	(7.7)	95,174	(6.0)	40,935	(5.0)
Dizziness	167,802	(3.0)	20,246	(1.8)	56,680	(2.8)	57,000	(3.6)	33,876	(4.1)
Dyspnoea	8,876	(0.2)	884	(0.1)	2,275	(0.1)	2,984	(0.2)	2,733	(0.3)
Foot problems	72,549	(1.3)	16,550	(1.5)	25,057	(1.2)	19,480	(1.2)	11,462	(1.4)
Fragility fracture	77,591	(1.4)	14,202	(1.3)	24,846	(1.2)	23,472	(1.5)	15,071	(1.8)
Hearing impairment	87,844	(1.6)	21,461	(1.9)	30,445	(1.5)	23,584	(1.5)	12,354	(1.5)
Heart failure	204,649	(3.7)	26,775	(2.4)	72,958	(3.6)	67,826	(4.3)	37,090	(4.5)
Heart valve disease	63,106	(1.1)	6,275	(0.6)	19,409	(0.9)	21,785	(1.4)	15,637	(1.9)
Housebound	7,505	(0.1)	383	(0.0)	1,605	(0.1)	2,425	(0.2)	3,092	(0.4)
Hypertension	534,683	(9.6)	155,411	(14.1)	210,972	(10.3)	120,439	(7.6)	47,861	(5.8)
Hypotension/syncope	145,568	(2.6)	15,357	(1.4)	47,552	(2.3)	50,788	(3.2)	31,871	(3.9)
Ischaemic heart disease	183,726	(3.3)	25,730	(2.3)	66,205	(3.2)	59,256	(3.8)	32,535	(3.9)
Memory and cognitive problems	159,259	(2.9)	28,491	(2.6)	57,893	(2.8)	46,837	(3.0)	26,038	(3.2)
Mobility and transfer problems	57,828	(1.0)	5,419	(0.5)	17,092	(0.8)	19,061	(1.2)	16,256	(2.0)
Osteoporosis	186,616	(3.4)	32,236	(2.9)	69,211	(3.4)	56,395	(3.6)	28,774	(3.5)
Parkinsonism and tremor	56,804	(1.0)	7,941	(0.7)	19,916	(1.0)	17,929	(1.1)	11,018	(1.3)
Peptic ulcer	478,326	(8.6)	119,408	(10.8)	190,347	(9.3)	119,868	(7.6)	48,703	(5.9)
Peripheral vascular disease	154,838	(2.8)	22,684	(2.1)	55,007	(2.7)	49,231	(3.1)	27,916	(3.4)
Polypharmacy	390,378	(7.0)	41,228	(3.7)	175,547	(8.6)	123,885	(7.9)	49,718	(6.0)
Requirement for care	7,505	(0.1)	383	(0.0)	1,605	(0.1)	2,425	(0.2)	3,092	(0.4)
Respiratory disease	316,297	(5.7)	87,074	(7.9)	113,826	(5.6)	78,121	(5.0)	37,276	(4.5)
Skin ulcer	64,565	(1.2)	12,784	(1.2)	20,585	(1.0)	18,426	(1.2)	12,770	(1.5)
Sleep disturbance	228,592	(4.1)	37,048	(3.4)	88,272	(4.3)	69,172	(4.4)	34,100	(4.1)
Social vulnerability	3,715	(0.1)	982	(0.1)	1,477	(0.1)	857	(0.1)	399	(0.0)
Thyroid disease	93,609	(1.7)	20,196	(1.8)	31,818	(1.6)	25,964	(1.6)	15,631	(1.9)
Urinary incontinence	34,040	(0.6)	3,850	(0.3)	10,452	(0.5)	11,372	(0.7)	8,366	(1.0)
Urinary system disease	127,639	(2.3)	28,289	(2.6)	43,337	(2.1)	35,058	(2.2)	20,955	(2.5)
Visual impairment	234,281	(4.2)	67,235	(6.1)	85,004	(4.1)	56,685	(3.6)	25,357	(3.1)
Weight loss and anorexia	86,738	(1.6)	9,521	(0.9)	26,602	(1.3)	28,735	(1.8)	21,880	(2.6)

**Table 2:** Adjusted predicted transition probabilities (%) of final status at 6 years after the initiation of follow-up according to baseline frailty status, age group, and sex

Sex	Age group	Baseline frailty status	Fit, %	Mild, %	Moderate, %	Severe, %	Death, %
All	All	Fit	58.5	21.9	5.6	1.2	12.7
		Mild	13.5	35.6	21.2	5.8	23.8
		Moderate	4.4	16.4	28.1	16.4	34.7
		Severe	2.6	6.4	15.7	26.2	49.1
Men	All	Fit	59.6	19.8	5.1	1.1	14.3
		Mild	13.2	34.2	18.9	5.4	28.5
		Moderate	3.8	14.4	24.3	14.1	43.3
		Severe	1.6	4.5	11.6	20.4	61.8
	50–64	Fit	78.0	13.7	2.4	0.5	5.4
		Mild	24.0	46.5	15.1	3.1	11.3
		Moderate	9.8	25.3	32.1	13.0	19.7
		Severe	3.7	7.0	19.0	32.8	37.5
	65–74	Fit	63.6	21.8	4.9	0.9	8.7
		Mild	18.8	42.9	20.4	4.5	13.3
		Moderate	6.7	21.4	33.1	15.9	22.9
		Severe	4.1	7.4	17.6	30.4	40.5
	75–84	Fit	40.1	25.4	8.6	2.2	23.8
		Mild	9.2	31.8	21.5	6.8	30.8
		Moderate	2.8	14.0	26.0	16.5	40.6
		Severe	1.2	4.4	12.8	23.5	58.1
	85+	Fit	18.8	14.8	5.8	1.6	59.0
		Mild	5.4	15.1	11.5	4.6	63.4
		Moderate	2.1	7.2	11.9	8.1	70.7
		Severe	0.9	3.0	6.2	9.6	80.4
Women	All	Fit	57.6	23.6	6.1	1.2	11.4
		Mild	13.7	36.5	22.8	6.2	20.8
		Moderate	4.7	17.5	30.2	17.6	29.9
		Severe	3.0	7.4	17.8	29.3	42.4
	50–64	Fit	78.7	16.1	2.6	0.4	2.2
		Mild	27.5	46.8	17.9	2.7	5.1
		Moderate	8.7	29.6	37.7	15.1	8.9
		Severe	2.7	8.8	24.4	41.7	22.4
	65–74	Fit	62.0	26.6	6.1	1.0	4.3
		Mild	16.8	45.7	25.3	5.7	6.5
		Moderate	4.5	23.5	40.7	20.8	10.5
		Severe	1.8	8.2	24.1	42.8	23.1
	75–84	Fit	43.4	30.4	10.1	2.2	13.9
		Mild	11.7	37.3	26.8	8.0	16.2
		Moderate	4.5	18.6	34.3	21.3	21.2
		Severe	3.0	8.3	21.4	36.4	31.0
	85+	Fit	21.7	16.4	6.2	1.8	53.9
		Mild	8.4	20.1	14.6	4.8	52.1
		Moderate	4.7	11.3	17.7	11.0	55.3
		Severe	3.4	6.0	11.5	16.4	62.6

Values are model-based predicted probabilities from weighted multinomial logistic regression. Predicted probabilities were averaged over the empirical covariate distribution using IPCW-based final weights. Age groups are shown directly; baseline frailty categories are fit, mild frailty, moderate frailty, and severe frailty.

**Table 3:** Adjusted odds ratios (95% confidence intervals) of sex for frailty status at 6 years after follow-up initiation by age and baseline frailty status

Age group	Baseline frailty status	Mild vs. Fit	Moderate vs. Fit	Severe vs. Fit	Death vs. Fit
50–64	Fit	1.16 (1.13, 1.20)	1.05 (0.98, 1.12)	0.84 (0.71, 0.99)	0.41 (0.39, 0.44)
	Mild	0.88 (0.82, 0.93)	1.03 (0.95, 1.12)	0.77 (0.66, 0.90)	0.39 (0.35, 0.43)
	Moderate	1.30 (1.06, 1.60)	1.31 (1.08, 1.60)	1.30 (1.04, 1.63)	0.50 (0.40, 0.63)
	Severe	1.68 (0.80, 3.49)	1.72 (0.88, 3.33)	1.70 (0.89, 3.25)	0.80 (0.42, 1.54)
65–74	Fit	1.25 (1.23, 1.28)	1.29 (1.24, 1.35)	1.12 (1.02, 1.23)	0.50 (0.48, 0.52)
	Mild	1.19 (1.15, 1.24)	1.40 (1.33, 1.46)	1.41 (1.32, 1.52)	0.55 (0.52, 0.58)
	Moderate	1.66 (1.47, 1.87)	1.86 (1.66, 2.08)	1.97 (1.75, 2.23)	0.70 (0.62, 0.79)
	Severe	2.45 (1.73, 3.46)	3.05 (2.22, 4.19)	3.14 (2.31, 4.28)	1.27 (0.93, 1.74)
75–84	Fit	1.11 (1.08, 1.14)	1.09 (1.04, 1.14)	0.91 (0.84, 0.99)	0.54 (0.52, 0.56)
	Mild	0.92 (0.88, 0.96)	0.97 (0.93, 1.02)	0.92 (0.86, 0.97)	0.41 (0.39, 0.43)
	Moderate	0.84 (0.76, 0.93)	0.83 (0.76, 0.91)	0.81 (0.74, 0.90)	0.33 (0.30, 0.36)
	Severe	0.77 (0.60, 0.98)	0.68 (0.54, 0.85)	0.63 (0.50, 0.78)	0.22 (0.17, 0.27)
85+	Fit	0.96 (0.90, 1.03)	0.92 (0.84, 1.02)	1.00 (0.85, 1.18)	0.79 (0.75, 0.83)
	Mild	0.86 (0.79, 0.94)	0.82 (0.74, 0.89)	0.68 (0.61, 0.76)	0.53 (0.49, 0.57)
	Moderate	0.70 (0.60, 0.82)	0.67 (0.57, 0.77)	0.60 (0.52, 0.70)	0.35 (0.30, 0.40)
	Severe	0.50 (0.36, 0.71)	0.47 (0.34, 0.65)	0.43 (0.32, 0.59)	0.20 (0.14, 0.27)

Values are adjusted odds ratios (95% confidence intervals) from multinomial logistic regression. The outcome reference category was fit at 6 years after follow-up initiation. The reference sex was men.

**Fig. 1:**
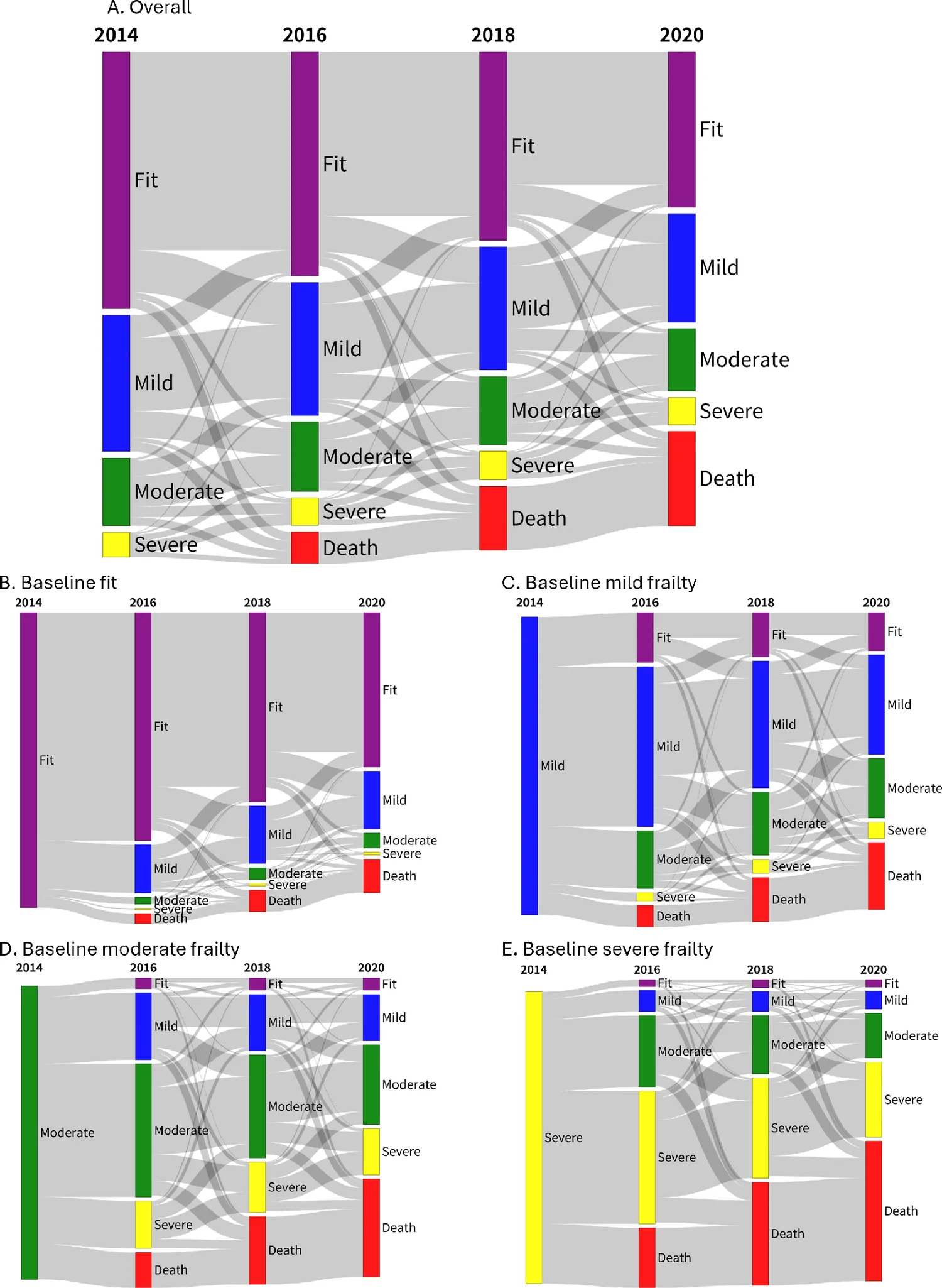
IPC-weighted frailty transition over 6 years overall and by baseline frailty status. Panel **A** shows the overall frailty trajectory. Panels **B**–**E** show frailty trajectories stratified by baseline frailty category: fit (**B**), mild frailty (**C**), moderate frailty (**D**), and severe frailty (**E**). IPC, inverse probability of censoring.

**Fig. 2:**
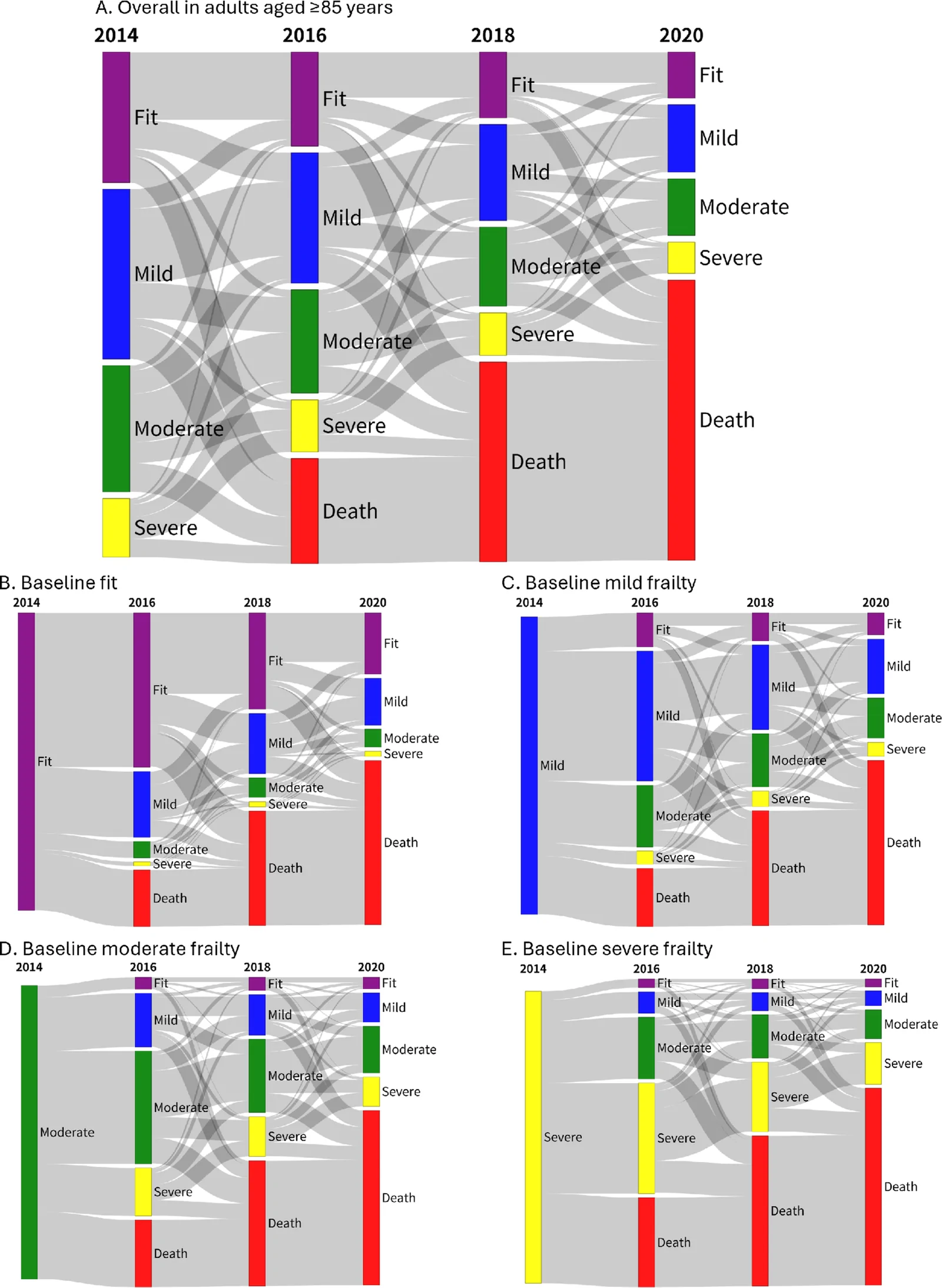
IPC-weighted frailty trajectories over 6 years among individuals aged ≥ 85 years overall and by baseline frailty status. Panel **A** shows frailty trajectory in individuals aged ≥85 years. Panels **B**–**E** show frailty trajectories stratified by baseline frailty category: fit (**B**), mild frailty (**C**), moderate frailty (**D**), and severe frailty (**E**). IPC, inverse probability of censoring.

### Overall frailty trajectories over 6 years

The loss to follow-up (insurance plan disenrollment) occurred in 37,622 (3.6%) in 2016, 28,515 (3.0%) in 2018, and 50,087 (5.9%) in 2020 (Supplementary Table 3). Stabilized weights showed little variability over time (Supplementary Table 4). After IPCW, among individuals who were fit at baseline, 58.5% remained fit at 6 years after follow-up initiation (Fig. 1; Table 2). Among those with mild frailty at baseline, the probability of transition to fit by 6 years was 13.5%. Corresponding probabilities among those with moderate and severe frailty at baseline were 4.4% and 2.6%, respectively. The weighted mortality proportions over 6 years among individuals who were fit and those with mild, moderate, and severe frailty at baseline were 12.7%, 23.8%, 34.7%, and 49.1%, respectively (Table 2). Individuals with severe frailty at baseline rarely transitioned to fit by 6 years and were more likely to die than those with milder frailty. The proportion transitioning to fit was 13.5% for mild frailty, 4.4% for moderate frailty, and 2.6% for severe frailty, whereas the proportion who died was 23.8%, 34.7%, and 49.1%, respectively.

### Frailty trajectories among individuals aged ≥ 85 years

Regarding the progression of frailty severity in the population aged 85 and older at baseline, the weighted probability of transition to fit by 6 years among those with mild, moderate, and severe frailty was as follows: 7.5% for mild frailty, 3.9% for moderate frailty, 2.7% for severe frailty (Fig. 2). The weighted mortality increased substantially over time, reaching 21.7% in 2016, 41.4% in 2018, and 58.2% in 2020 (Fig. 2). In the sex-stratified analysis, mortality proportions were consistently higher in men than in women over time: 26.3% versus 19.6% in 2016, 48.6% versus 38.1% in 2018, and 65.9% versus 54.7% in 2020 (Table 2).

Among individuals who were initially fit, the proportion of those who remained fit during 6-year follow-up was 20.8%. For those with mild frailty at baseline, the probability of transition to fit within six years was 7.5%. Similarly, for those with moderate and severe frailty at baseline, the probability of transition to fit within six years were 3.9% and 2.7%, respectively (Fig. 2). Additionally, mortality during the 6-year follow-up was high regardless of the baseline frailty severity. The cumulative death proportion within six years was 55.6% for fit, 55.6% for mild frailty, 59.9% for moderate frailty, and 67.9% for severe frailty (Fig. 2). Death was not always preceded by severe frailty as measured two years prior, but could occur from any health state within this age group.

### Sex- and age-specific frailty trajectories

Overall, in the IPC-weighted analysis, among the individuals with severe frailty at baseline, mortality increased substantially over time, reaching 42.4% in women and 61.8% in men by 6 years (Table 2 and Supplementary Figs. 5 A and 5B). By baseline frailty category, the 6-year mortality proportions in women versus men were 11.4% versus 14.3% for fit, 20.8% versus 28.5% for mild frailty, 29.9% versus 43.3% for moderate frailty, and 42.4% versus 61.8% for severe frailty (Table 2 and Supplementary Figs. 6 A and 6B). The corresponding probabilities of transition to fit among those with mild, moderate, and severe frailty at baseline were 13.7% versus 13.2%, 4.7% versus 3.8%, and 3.0% versus 1.6% in women and men, respectively (Table 2 and Supplementary Figs. 6 A and 6B).

Among individuals aged 65–74 years, women had lower predicted probabilities of death at 6 years than men across all baseline frailty categories. Among those who were fit, mildly frail, moderately frail, and severely frail at baseline, death occurred in 4.3% of women versus 8.7% of men, 6.5% versus 13.3%, 10.5% versus 22.9%, and 23.1% versus 40.5%, respectively. In addition, among those who were fit at baseline, mild and moderate frailty at 6 years were more frequent in women than in men (26.6% vs. 21.8% and 6.1% vs. 4.9%, respectively) (Table 2). Similar patterns were observed among those with mild, moderate, or severe frailty at baseline. Among individuals aged 65–74 years who were fit at baseline, women had lower odds of death and higher odds of non-fatal frailty states relative to fit at 6 years than men. The odds ratios for women versus men were 1.25 (95% CI, 1.23–1.28) for mild frailty, 1.29 (95% CI, 1.24–1.35) for moderate frailty, 1.12 (95% CI, 1.02–1.23) for severe frailty, and 0.50 (95% CI, 0.48–0.52) for death (Table 3).

Among individuals aged ≥ 85 years, the weighted probability of transition (women versus men) to fit by 6 years among those with mild, moderate, and severe frailty was as follows: 8.4% versus 5.4%, 4.7% versus 2.1%, and 3.4% versus 0.9%, respectively (Table 2). Women had lower predicted probabilities of death than men across all baseline frailty categories, but in contrast to those aged 65–74 years, women were also more likely to remain in or transition to fit. Among those who were fit at baseline, the predicted probability of fit at 6 years was 21.7% in women versus 18.8% in men, while death occurred in 53.9% versus 59.0%, respectively. Similar differences were observed among those with mild, moderate, or severe frailty at baseline, for whom death occurred in 52.1% versus 63.4%, 55.3% versus 70.7%, and 62.6% versus 80.4%, respectively (Table 2). Among those who were fit at baseline, women generally had similar odds of non-fit frailty states and lower odds of death than men; the odds ratios were 0.96 (95% CI, 0.90–1.03) for mild frailty, 0.92 (95% CI, 0.84–1.02) for moderate frailty, 1.00 (95% CI, 0.85–1.18) for severe frailty, and 0.79 (95% CI, 0.75–0.83) for death, respectively (Table 3). By comparison, among those with mild, moderate, or severe frailty at baseline, women had lower odds of both non-fatal frailty states and death than men.

## Discussion

We evaluated the trajectory of frailty over 6 years in a large population aged ≥ 50 years using eFI derived from a regional administrative claims database in Japan. Notably, 41.5% of individuals who were fit at baseline transitioned to a frailty state or died during the 6-year follow-up. Severe frailty was associated with limited recovery and high mortality; only 2.6% reached fit status and 9.0% reached fit or mild frailty at 6 years, whereas 91.0% died or were in moderate-to-severe frailty. Moreover, sex differences in frailty trajectories varied across age groups. Among fit individuals aged 65–74 years, women had lower mortality than men and were more likely to be alive with non-fatal frailty states at 6 years. In contrast, among those aged ≥ 85 years, death was the dominant final state, although women had lower mortality and higher probabilities of being in the fit category than men. These findings highlight severe frailty as an advanced high-risk state, in which recovery to fit or mild status is uncommon and death constitutes an important part of the trajectory.

The demographics and baseline eFI status in our study showed comparable findings to the study originally reported the development of the eFI [9]. The eFI distribution exhibited a pronounced right skewness, which further shifted towards the right throughout the study period. This observed shift suggests a collective advancement of frailty within the study population. Walsh et al. demonstrated that frailty worsened over time for all frailty categories [28], and the results of our study showed a similar trend.

In the overall cohort, we found that the proportion of individuals who maintained their baseline frailty category even after six years was 58.5% for those with a baseline of “fit” and 26.2% for those with a baseline of “severe frailty”. Furthermore, among those with a baseline of “fit”, 28.7% individuals transitioned to more frail categories (mild, moderate, or severe) after six years, with 12.7% of them dying. In contrast, among those with a baseline of “severe frailty”, 49.1% died after six years. This finding is consistent with previous research using frailty phenotype [13]. Gill et al. reported that during a 54-month follow-up, the proportion of individuals experiencing at least 1 transition between any 2 of the 3 frailty states was 58%, and worsening of frailty was more common than improvement.

As reported in previous research, we observed the highest proportion of death among individuals with severe frailty at baseline. A distinct pattern was observed among individuals aged ≥ 85 years, in whom death was common across all baseline frailty categories. This may be in part be explained by the limited ability of our eFI measurement method to capture the rapid deterioration of frailty over periods shorter than two years, more frequent especially in older adults. According to prior research [15], the trajectory of eFI over a 5-year period until death showed that the acceleration of deterioration was not steady. Thus, it may be necessary in the future to consider altering the length of the frailty assessment period and the timing of assessments.

In the present study, we found that the association between sex and 6-year frailty status appeared to differ across age groups, suggesting an interaction between sex and age group. Among individuals aged 65–74 years, women were less likely than men to die over 6 years, and were more likely to be in non-fatal frailty states rather than remaining fit. In contrast, among individuals aged ≥ 85 years, women had lower mortality than men and were more likely to remain in or transition to fit. Among those who were fit at baseline, women had similar probabilities of non-fit frailty states but lower mortality than men. Among those with baseline frailty, women had lower mortality and higher probabilities of fit and non-fatal frailty states than men; however, because fit was more common in women, the relative odds of both non-fatal frailty states and death versus fit were lower in women than in men. Although prior studies mainly focused on independent predictors rather than the interaction between age and sex, previous studies on sex differences in frailty worsening have been inconsistent [29, 30]. A longitudinal study has suggested that women may have a higher likelihood of frailty progression than men [29], whereas another has reported more frequent worsening in men [30]. The lower mortality observed in women may partly reflect the well-described women’s survival advantage, which has been attributed to biological factors such as sex hormones and their effects on cardiovascular risk [31]. However, these mechanisms alone are unlikely to fully explain the sex differences observed at very old ages. It is also possible that men are more likely to be at higher risk of death [32], whereas women more often survive with chronic conditions that are disabling and associated with greater morbidity [33–35]. These mechanisms may collectively contribute to the sex-specific patterns of frailty progression and mortality observed in our study.

Our finding of limited transition from severe frailty to the fit category underscores the importance of risk stratification, early intervention for older adults with relatively mild frailty, and strategies to mitigate further decline among those with severe frailty. Frailty is increasingly conceptualized not as an inevitably progressive condition, but as a dynamic state characterized by potential transitions between levels of vulnerability [36]. Prior evidence suggests that frailty may be influenced by potentially modifiable factors and interventions. Multicomponent interventions that include components such as exercise [37–40], cognitive training [39], nutrition [37, 38, 40], an antidepressant program [37], deprescribing [37, 40], and avoiding home hazards [37] may be candidate strategies for improving overall survival [37] and preserving mobility [38, 39] among individuals with non-severe frailty [37]. Practical and easily implementable interventions may also be important for promoting frailty reversal in older adults with non-severe frailty [41]. However, these trials generally included participants with relatively mild frailty or pre-frailty, and their applicability to older adults with more advanced frailty remains uncertain. For older adults with severe frailty, individualized, assessment-based approaches such as a comprehensive geriatric assessment may be more appropriate and beneficial for health outcomes in routine medical care settings [42].

In Japan, national long-term care prevention strategies emphasize community-based integrated care initiatives aimed at maintaining functional capacity and preventing disability [43]. Within this policy context, our trajectory analysis offers a population-level description of frailty transitions under routine care conditions. In our cohort, reversal from severe frailty to fit status was uncommon (2.6%). One possible interpretation is that once functional deficits accumulate beyond a certain threshold, opportunities for meaningful improvement may become more limited. However, because our analyses were based on administrative claims data and did not include direct information on participation in structured interventions or medication review processes, this explanation remains speculative. Further studies integrating clinical and program-level data would be needed to clarify the mechanisms underlying frailty transitions in routine practice. Since the prevalence of diseases and conditions used in eFI calculation varies by sex and age, even with the same frailty category, the deficit profiles may differ. Clarifying the differences in the quality of frailty could lead to a more appropriate allocation of healthcare resources and clearer targeting of interventions. Fogg et al. found that as frailty increases within age groups, so does the cost of care, emphasizing the importance of early intervention to maintain or improve the frailty category [44]. More research is needed for investigating the association between individual characteristics and improvement across different age groups and frailty categories.

### Limitations

Several limitations should be considered when interpreting the results. First, we attempted to account for differences in disenrollment by frailty status using IPCW estimated from a model including age, sex, and time-varying frailty status. However, residual bias due to unmeasured factors related to disenrollment may remain. Second, the study population is regional, focusing on Shizuoka Prefecture in Japan. The calculation of the eFI scores is based on data from subscribers to Japan’s National Health Insurance and the Late-Stage Elderly Medical Care System. Consequently, the generalizability of the findings to other regions, populations in Japan, or individuals overseas is not guaranteed. Third, there is currently no unified consensus on the definition of frailty, and the validation of frailty as measured by eFI against physical frailty scale, such as the CHS criteria (the current gold standard), has not yet been conducted in Japan. Fourth, the absence of falls from the eFI components may have resulted in slight underestimation of frailty severity and potentially attenuated observed transitions or mortality associations. However, prior methodological work suggests that frailty indices constructed according to established standard criteria have been shown to demonstrate stable distributional properties despite variation in the specific deficits included [7, 45]. Fifth, particularly for chronic or non-acute conditions, a recorded code may reflect an ongoing condition from an earlier period rather than a newly active problem, and the extent to which such carry-forward occurred could not be directly evaluated using claims data alone. Conversely, the absence of a code does not necessarily indicate the true absence of a condition, because coding depends on healthcare utilization, clinical recognition, and claims documentation practices. As a result, some deficits may have been misclassified. Finally, the SKDB does not include detailed information on exercise, nutrition, medication review, deprescribing, social support, or participation in structured interventions. Therefore, our study cannot determine whether these factors explain the observed transition patterns or whether specific interventions would alter frailty transitions. Future studies linking clinical, behavioral, social, and program-level data are needed to identify modifiable determinants of frailty improvement and prevention of progression, particularly among older adults with severe frailty.

## Conclusions

This longitudinal study, including a substantial number of the oldest-old, demonstrates the limited reversibility of severe frailty and substantial heterogeneity in sex differences across age groups. These findings support age- and sex-tailored prevention and care strategies and may inform healthcare policy planning in aging societies.

## Supplementary Information

Below is the link to the electronic supplementary material.

Supplementary Material 1

## Data Availability

The data used in this study are not publicly available due to contractual agreements with Shizuoka Prefecture. Access to the dataset is restricted in accordance with the terms and conditions set by the Shizuoka Prefectural Government.
