# Supplementary material for: Trajectory of frailty among the older people in a community setting: A cohort study from the Shizuoka Kokuho Database
Source: Aging Clin Exp Res. 2026 Jun 14;38(1):178. doi: 10.1007/s40520-026-03434-z (PMC13490216; doi:10.1007/s40520-026-03434-z)
Supplement: Supplementary file 1 [file 40520_2026_3434_MOESM1_ESM.docx]

**Supplementary Data**

**Supplement to:** Trajectory of frailty among the older people in a community setting: a cohort study from the Shizuoka Kokuho Database

**Table of contents**

**Supplementary Figure 1.** Flow diagram of the enrolment of the study cohort

**Supplementary Table 1.** List of deficits included in the electronic Frailty Index (eFI)

**Supplementary Figure 2.** Timeline of the 12-month look-back period and frailty assessment periods

**Supplementary Figure 3.** Distribution of electronic Frailty Index (eFI) scores at each assessment time point

**Supplementary Table 2.** Baseline characteristics among individuals aged ≥85 years

**Supplementary Figure 4.** Crude frailty transitions over 6 years

**Supplementary Table 3.** Cumulative numbers of deaths and loss to follow-up by baseline frailty status and assessment year

**Supplementary Table 4.** Weight diagnostics for IPCW used in Sankey diagrams

**Supplementary Figure 5A.** IPC-weighted frailty transitions in women

**Supplementary Figure 5B.** IPC-weighted frailty transitions in men

**Supplementary Figure 6A.** IPC-weighted frailty transitions by baseline frailty status in women

**Supplementary Figure 6B.** IPC-weighted frailty transitions by baseline frailty status in men

**Supplementary Figure 1.** Flow diagram of the enrolment of the study cohort

**Supplementary Table 1.** List of deficits included in the electronic Frailty Index (eFI)

| Deficits | |
| --- | --- |
| Activity limitation | Ischaemic heart disease |
| Anaemia and haematinic deficiency | Memory and cognitive problems |
| Arthritis | Mobility and transfer problems |
| Atrial fibrillation | Osteoporosis |
| Cerebrovascular disease | Parkinsonism and tremor |
| Chronic kidney disease | Peptic ulcer |
| Diabetes | Peripheral vascular disease |
| Dizziness | Polypharmacy |
| Dyspnoea | Requirement for care |
| Falls | Respiratory disease |
| Foot problems | Skin ulcer |
| Fragility fracture | Sleep disturbance |
| Hearing impairment | Social vulnerability |
| Heart failure | Thyroid disease |
| Heart valve disease | Urinary incontinence |
| Housebound | Urinary system disease |
| Hypertension | Visual impairment |
| Hypotension/syncope | Weight loss and anorexia |

**Supplementary Figure 2.** Timeline of the 12-month look-back period and frailty assessment periods

**Supplementary Figure 3.** Distribution of electronic Frailty Index (eFI) scores at each assessment time point

**Supplementary Table 2.** Baseline characteristics among individuals aged ≥85 years

| Characteristics |  |  | Frailty category | |  |  |  |  |  |  |
| --- | --- | --- | --- | --- | --- | --- | --- | --- | --- | --- |
|  | Total, n (%) | | Fit, n (%) | | Mild frailty, n (%) | | Moderate frailty, n (%) | | Severe frailty, n (%) | |
|  | 156,019 | (100) | 41,867 | (100) | 54,618 | (100) | 40,673 | (100) | 18,861 | (100) |
| Women, n (%) | 107,624 | (69.0) | 28,052 | (67.0) | 37,579 | (68.8) | 28,514 | (70.1) | 13,273 | (70.4) |
| Deficits in the electronic Frailty Index, n (%) |  |  |  |  |  |  |  |  |  |  |
| Activity limitation | 16,136 | (1.3) | 668 | (0.6) | 3,471 | (0.9) | 5,617 | (1.2) | 6,380 | (2.1) |
| Anaemia and haematinic deficiency | 39,385 | (3.2) | 3,096 | (3.0) | 11,807 | (3.0) | 14,292 | (3.2) | 10,190 | (3.4) |
| Arthritis | 56,787 | (4.5) | 5,382 | (5.1) | 18,908 | (4.8) | 20,531 | (4.5) | 11,966 | (4.0) |
| Atrial fibrillation | 34,643 | (2.8) | 2,207 | (2.1) | 9,876 | (2.5) | 13,104 | (2.9) | 9,456 | (3.2) |
| Cerebrovascular disease | 59,691 | (4.8) | 5,360 | (5.1) | 19,082 | (4.9) | 21,719 | (4.8) | 13,530 | (4.5) |
| Chronic kidney disease | 15,162 | (1.2) | 829 | (0.8) | 3,731 | (0.9) | 5,594 | (1.2) | 5,008 | (1.7) |
| Diabetes | 70,645 | (5.7) | 8,162 | (7.8) | 24,425 | (6.2) | 24,143 | (5.3) | 13,915 | (4.7) |
| Dizziness | 38,419 | (3.1) | 1,347 | (1.3) | 9,061 | (2.3) | 15,705 | (3.5) | 12,306 | (4.1) |
| Dyspnoea | 2,427 | (0.2) | 105 | (0.1) | 412 | (0.1) | 897 | (0.2) | 1,013 | (0.3) |
| Foot problems | 14,510 | (1.2) | 1,179 | (1.1) | 4,361 | (1.1) | 5,162 | (1.1) | 3,808 | (1.3) |
| Fragility fracture | 25,221 | (2.0) | 2,549 | (2.4) | 7,255 | (1.8) | 8,885 | (2.0) | 6,532 | (2.2) |
| Hearing impairment | 15,142 | (1.2) | 1,509 | (1.4) | 4,215 | (1.1) | 5,499 | (1.2) | 3,919 | (1.3) |
| Heart failure | 61,018 | (4.9) | 4,359 | (4.2) | 18,901 | (4.8) | 23,204 | (5.1) | 14,554 | (4.9) |
| Heart valve disease | 15,819 | (1.3) | 616 | (0.6) | 3,615 | (0.9) | 6,082 | (1.3) | 5,506 | (1.8) |
| Housebound | 2,662 | (0.2) | 75 | (0.1) | 417 | (0.1) | 907 | (0.2) | 1,263 | (0.4) |
| Hypertension | 111,491 | (8.9) | 15,763 | (15.1) | 42,605 | (10.8) | 35,553 | (7.9) | 17,570 | (5.9) |
| Hypotension/syncope | 34,506 | (2.8) | 1,081 | (1.0) | 7,674 | (2.0) | 14,132 | (3.1) | 11,619 | (3.9) |
| Ischaemic heart disease | 45,254 | (3.6) | 2,601 | (2.5) | 13,236 | (3.4) | 17,577 | (3.9) | 11,840 | (4.0) |
| Memory and cognitive problems | 46,478 | (3.7) | 5,930 | (5.7) | 15,121 | (3.8) | 15,382 | (3.4) | 10,045 | (3.4) |
| Mobility and transfer problems | 16,136 | (1.3) | 668 | (0.6) | 3,471 | (0.9) | 5,617 | (1.2) | 6,380 | (2.1) |
| Osteoporosis | 48,681 | (3.9) | 4,105 | (3.9) | 15,842 | (4.0) | 17,770 | (3.9) | 10,964 | (3.7) |
| Parkinsonism and tremor | 11,228 | (0.9) | 606 | (0.6) | 2,976 | (0.8) | 4,272 | (0.9) | 3,374 | (1.1) |
| Peptic ulcer | 91,851 | (7.3) | 8,051 | (7.7) | 33,201 | (8.4) | 33,281 | (7.3) | 17,318 | (5.8) |
| Peripheral vascular disease | 30,414 | (2.4) | 1,512 | (1.4) | 8,084 | (2.1) | 11,855 | (2.6) | 8,963 | (3.0) |
| Polypharmacy | 94,477 | (7.6) | 5,267 | (5.0) | 36,066 | (9.2) | 35,373 | (7.8) | 17,771 | (5.9) |
| Requirement for care | 2,662 | (0.2) | 75 | (0.1) | 417 | (0.1) | 907 | (0.2) | 1,263 | (0.4) |
| Respiratory disease | 64,101 | (5.1) | 6,770 | (6.5) | 20,931 | (5.3) | 22,630 | (5.0) | 13,770 | (4.6) |
| Skin ulcer | 17,345 | (1.4) | 1,528 | (1.5) | 4,860 | (1.2) | 6,076 | (1.3) | 4,881 | (1.6) |
| Sleep disturbance | 49,802 | (4.0) | 3,106 | (3.0) | 15,751 | (4.0) | 18,965 | (4.2) | 11,980 | (4.0) |
| Social vulnerability | 245 | (0.0) | 18 | (0.0) | 75 | (0.0) | 74 | (0.0) | 78 | (0.0) |
| Thyroid disease | 15,827 | (1.3) | 1,100 | (1.1) | 4,102 | (1.0) | 5,738 | (1.3) | 4,887 | (1.6) |
| Urinary incontinence | 9,911 | (0.8) | 554 | (0.5) | 2,440 | (0.6) | 3,680 | (0.8) | 3,237 | (1.1) |
| Urinary system disease | 27,672 | (2.2) | 2,243 | (2.1) | 7,691 | (2.0) | 9,947 | (2.2) | 7,791 | (2.6) |
| Visual impairment | 42,151 | (3.4) | 5,205 | (5.0) | 14,009 | (3.6) | 14,578 | (3.2) | 8,359 | (2.8) |
| Weight loss and anorexia | 21,979 | (1.8) | 1,045 | (1.0) | 5,032 | (1.3) | 8,134 | (1.8) | 7,768 | (2.6) |

**Supplementary Figure 4.** Crude frailty transitions over 6 years

**Supplementary Table 3.** Cumulative numbers of deaths and loss to follow-up by baseline frailty status and assessment year

|  | | Cumulative number of deaths | | Cumulative number of participants lost to follow-up | |
| --- | --- | --- | --- | --- | --- |
|  |  | N | (%) | N | (%) |
| 2016 | All | 66,108 |  | 37,622 |  |
|  | Fit | 18,214 | (27.6) | 28,755 | (76.4) |
|  | Mild | 20,579 | (31.1) | 6,069 | (16.1) |
|  | Moderate | 16,759 | (25.4) | 2,120 | (5.6) |
|  | Severe | 10,556 | (16.0) | 678 | (1.8) |
| 2018 | All | 131,643 |  | 66,137 |  |
|  | Fit | 39,084 | (29.7) | 50,174 | (75.9) |
|  | Mild | 42,157 | (32.0) | 11,124 | (16.8) |
|  | Moderate | 32,241 | (24.5) | 3,736 | (5.6) |
|  | Severe | 18,161 | (13.8) | 1,103 | (1.7) |
| 2020 | All | 195,018 |  | 116,224 |  |
|  | Fit | 60,270 | (30.9) | 84,064 | (72.3) |
|  | Mild | 63,427 | (32.5) | 23,021 | (19.8) |
|  | Moderate | 46,647 | (23.9) | 7,286 | (6.3) |
|  | Severe | 24,674 | (12.7) | 1,853 | (1.6) |

**Supplementary Table 4.** Weight diagnostics for IPCW used in Sankey diagrams

| Stage | N used in Sankey | Sum of weights | Mean | SD | Min | Median | Max |
| --- | --- | --- | --- | --- | --- | --- | --- |
| 2014 | 1,003,041 | 1,003,056.0 | 1 | 0.008 | 0.967 | 1.002 | 1.0083 |
| 2016 | 1,003,041 | 1,003,056.0 | 1 | 0.008 | 0.967 | 1.002 | 1.0083 |
| 2018 | 908,418 | 908,478.0 | 1 | 0.008 | 0.963 | 1.001 | 1.0101 |
| 2020 | 792,796 | 792,887.0 | 1 | 0.008 | 0.962 | 1.001 | 1.0126 |

**Supplementary Figure 5A.** IPC-weighted frailty transitions in women

**Supplementary Figure 5B.** IPC-weighted frailty transitions in men

**Supplementary Figure 6A.** IPC-weighted frailty transitions by baseline frailty status in women

**Supplementary Figure 6B.** IPC-weighted frailty transitions by baseline frailty status in men
